# Preparation and Characterization of PVA/PDDA/Nano-Zirconia Composite Anion Exchange Membranes for Fuel Cells

**DOI:** 10.3390/polym11091399

**Published:** 2019-08-26

**Authors:** Asep Muhamad Samsudin, Viktor Hacker

**Affiliations:** 1 Institute of Chemical Engineering and Environmental Technology, Graz University of Technology, 8010 Graz, Austria; 2 Department of Chemical Engineering, Diponegoro University, Jawa Tengah 50275, Indonesia

**Keywords:** anion exchange membranes, poly(vinyl alcohol), poly(diallyldimethylammonium chloride), zirconia, fuel cells

## Abstract

Anion exchange membranes (AEMs) contribute significantly to enhance the performance and efficiency of alkaline polymer electrolyte fuel cells (APEFCs). A sequence of composite anion exchange membranes (AEMs) consisting of poly(vinyl alcohol) (PVA), poly(diallyldimethylammonium chloride) (PDDA), and nano-zirconia (NZ) has been prepared by a solution casting technique. The effect of zirconia mass ratio on attribute and performance of composite AEMs was investigated. The chemical structures, morphology, thermal, and mechanical properties of AEMs were characterized by FTIR, SEM, thermogravimetric analysis, and universal testing machine, respectively. The performance of composite AEMs was verified using water uptake, swelling degree, ion-exchange capacity, and OH^−^ conductivity measurement. The nano-zirconia was homogeneously dispersed in the PVA/PDDA AEMs matrix. The mechanical properties of the composite AEMs were considerably enhanced with the addition of NZ. Through the introduction of 1.5 wt.% NZ, PVA/PDDA/NZ composite AEMs acquired the highest hydroxide conductivity of 31.57 mS·cm^−1^ at ambient condition. This study demonstrates that the PVA/PDDA/NZ AEMs are a potential candidate for APEFCs application.

## 1. Introduction

The depletion of fossil energy and the rise of environmental issues have led to high demand for eco-clean, efficient, and sustainable alternative energy. Among developed alternative energy, fuel cells are regarded as a potential candidate to address these issues. 

Polymer electrolyte fuel cells (PEFCs) have been intensively studied due to their significant performance and prospects, corresponding to other types of fuel cells. However, the commercialization process has been impeded by several issues, for instance, considerable cost due to a platinum catalyst in the electrode layer, sluggish reaction kinetics in an acidic environment, fuel crossover, and complex water management [1,2]. Recently, the development of APEFCs has attracted more attention. Occasioned by the use of anion-exchange membranes in place of proton-exchange membranes, OH^−^ transport occurs instead of H^+^ ions, which then affords several benefits in comparison with the PEMFCs. These include high oxygen reduction rate in the cathode, use of inexpensive and non-noble catalysts (e.g., silver, nickel, and palladium), low fuel permeability due to the opposite direction with hydroxide ion, and excellent corrosion resistance in the alkaline condition [3,4]. Thus, AEMs contribute significantly to improving the performance and efficiency of APEFCs [5]. 

Over the last few years, aside from well-known quaternized-based polymers AEMs such as polysulfone [5,6], polystyrene [7,8], poly(vinyl alcohol) [9,10], and poly (2,6-dimethyl-1,4-phenylene oxide), new quaternized polymers AEMs including poly(butylimidazolium) [11], poly(aryl ether oxadiazole) [12], poly(arylene ether sulfone) [13], poly(arylene ether nitrile) [14], poly(arylene ether ketone sulfone) [15], and radiation-grafted poly(ethylene-co-tetrafluoroethylene) [16] have been successfully fabricated and characterized. Nevertheless, these type of polymers are generally costly, and require a complex quaternization process, as well as frequently using toxic, even carcinogenic, substances [17]. 

To improve the performance of AEMs, many approaches have been developed, including the design of a comb-shaped structure [18] or the extension of flexible spacers between the quaternary ammonium groups and the polymer backbone [19,20] with the introduction of multiple functional groups [21], plasticizer [22], and ionic liquid [23]. At present, inorganic fillers such as alkoxysilane, oxides (e.g., SiO_2_ [24], Al_2_O_3_ [25], TiO_2_ [26], and ZrO_2_ [27]), and nano-carbon (e.g., graphene [6], CNT [28]) are frequently incorporated into the membrane matrix to improve the mechanical, thermal, chemical, and electrochemical properties [4]. Amongst them, ZrO_2_ has been widely used as an additive for AEMs fabrication because of good hydrophilicity and chemical stability in both the acidic and alkaline condition. In particular, nano-sized ZrO_2_ provides high surface area and activity as well as excellent chemical and thermal stability [29,30].

In this study, the composite AEMs based on PVA, PDDA, and NZ blends have been successfully prepared and characterized. PVA is a polyhydroxy-type polymer which possesses the excellent film-forming characteristic, hydrophilic, non-toxicity, and high density of reactive chemical functional groups which are beneficial for irradiation, chemical, as well as thermal cross-linking [31]. PDDA contains quaternary ammonium functional groups that are able to offer OH^−^ as charge carriers [32]. Furthermore, the cyclic structures of PDDA establish large steric hindrance, impeding the quaternary ammonium functional groups degradation through S_N_2 nucleophilic substitution in an alkaline environment [33]. PDDA also has a water-soluble characteristic and is environmentally friendly. In order to improve the characteristics of AEM, especially electrochemical and mechanical properties, nano-zirconia was incorporated into the polymer solution. The straightforwardness of the blending method permits the AEMS to be cost-efficient, offers to potentially concatenate the beneficial nature of respective blend material. Furthermore, the effect of zirconia mass ratio was investigated in composite PVA/PDDA/NZ membrane fabrication.

## 2. Materials and Methods 

### 2.1. Membrane Preparation 

**Figure 1:**
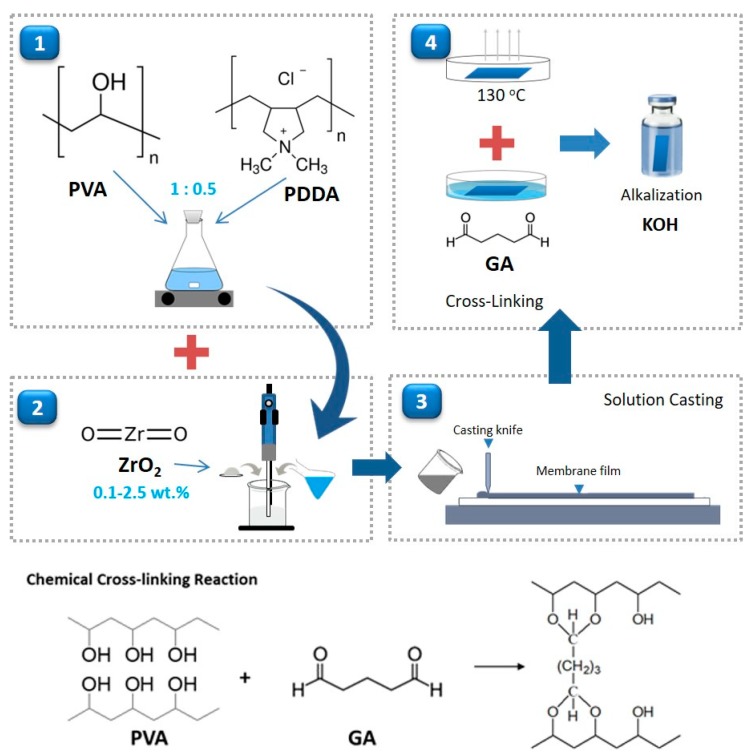
Procedure of the PVA//PDDA/NZ AEMs preparation.

Composite AEMs were fabricated using a solution casting technique. Typically, 20 g of PVA (*M_w_* = 31,000–50,000 g·mol^−1^, 98–99% hydrolyzed, Sigma-Aldrich, Vienna, Austria) was dissolved in 180 g of ultra-pure water at 80–90 °C while continuously stirring to obtain 10 wt.% PVA. Afterward, 100 g of 10 wt.% PDDA solution (20 wt.% aqueous solution, *M_w_* = 400,000–500,000, Sigma-Aldrich, Vienna, Austria) was then blended with 200 g of aforementioned PVA solution, resulting in PVA/PDDA with a weight ratio of 1:0.5. An appropriate amount of nano-zirconia (0–2.5 wt.%) (<100 nm, SA ≥ 25 m^2^/g, Sigma-Aldrich, Vienna, Austria) was introduced to PVA/PDDA solution under stirring and ultra-sonication for one hour. The resulting solutions were cast onto the glass surface with Elcometer 4340 (elcometer, Michigan, IN, USA) automatic film applicator and evaporated under ambient conditions for 24 h. Afterward, the dried membranes were peeled from the substrate. Figure 1 illustrates the procedure of PVA//PDDA/NZ AEMs preparation.

### 2.2. Cross-Linking 

In favor of restraining the swelling-tendency and improving the mechanical properties of AEMs, combination cross-linking of the polymer chains was introduced to the membrane preparation. The membranes were annealed at 130 °C for an hour to promote physical cross-linking and immersed in a cross-linker solution (10 wt.% glutaraldehyde (GA), 0.2 wt.% HCl in acetone solvent) to induce chemical cross-linking between PVA chains. 

### 2.3. Structure Characterization

FTIR spectra were obtained using IR-spectrometer (Bruker ALPHA, Billerica, MA, USA) with a wavenumber range of 500–4000 cm^−1^. The surface morphology of membranes was recorded using an SEM measurement (Carl Zeiss Supra 40, Oberkochen, Germany). Thermogravimetric analysis (TGA) was accomplished with an STA 449 C apparatus (NETZSCH, Selb, Germany). The membrane samples were loaded into the alumina sample holder following heating at a rate of 10 °C min^−1^ from temperatures of 25 °C to 600 °C under N_2_ gas flow of 40 ml/min. The mechanical properties of the tested samples at ambient condition were measured using an AGS-X universal testing machine (Shimadzu, Tokyo, Japan) at the strain rate of 5 mm·min^−1^. The sample width and length of the AEMs samples were 15 and 20 mm, respectively. 

### 2.4. Swelling Properties

Water uptake (WU) and swelling degree (SD) of the tested AEMs were determined by measuring the alteration in weight and volume of the membrane before and after water immersion. The tested samples were immersed in ultra-pure water at ambient condition for 24 h. The weight and volume of the swelling membranes were determined immediately after removing the surface water gently. The WU and SD of the AEMs were calculated using Equations (1) and (2), respectively.

(1) $$\mathrm{WU}=\frac{W_{w}\;-\;W_{d}}{W_{d}}\;\times \;100\%$$

(2) $$\mathrm{SD}=\frac{V_{w}\;-\;V_{d}}{V_{d}}\;\times \;100\%$$

where *W_d_* and *W_w_* are the weight of the respective dry and wet AEMs. *V_d_* and *V_w_* are the volume of the dry and wet tested AEMs, respectively.

### 2.5. Ion Exchange Capacity (IEC)

The IEC was assessed using a back-titration technique. The membrane samples were immersed in 1.0 M KOH solution for 24 h in order to transform the Cl^−^ into the OH^−^ form membrane. Afterward, the membrane was soaked with ultra-pure water to wipe the KOH residue for 24 h. Then, the membrane was equilibrated with a 0.1 M HCl solution for 24 h. The IEC was calculated from the decrease in acid value resulting from the titration. The IEC value was determined by Equation (3):

(3) $$\mathrm{IEC}=\frac{\left(V_{b}-V_{m}\right).C_{\mathrm{HCl}}}{w_{d}}$$

where *V_b_* is the consumed NaOH volumes of the blank sample, *V_m_* is the consumed NaOH volumes of the AEMs sample, *C*_HCl_ is the concentration of HCl solution, and *w_d_* is the weight of the dry AEMs sample.

### 2.6. OH^−^ Conductivity 

The OH^−^ conductivity was determined by an AC impedance method using Gamry Reference 600 potentiostat [34]. The membrane at a size of 2.5 × 1.0 cm was alkalized in a 1.0 M KOH solution for 24 h, washed, then immersed in ultra-pure water for 24 h. Impedance was measured over a frequency range of 0.1 Hz to 10 kHz (50 mV amplitude) with the samples placed into the four-electrode configuration of the conductivity clamp (Bekktech BT110 LLC, Scribner Associates, Southern Pines, NC, USA) that was immersed in ultra-pure water at ambient temperatures. The measured resistance *R_tot_* of the membrane was obtained from the intercept of the Nyquist curve with Z_real_ axis. Afterward, the membrane resistance (*R_m_*) and OH^−^ conductivity was calculated according to Equations (4) and (5), respectively.

(4) $$\frac{1}{R_{m}}=\frac{1}{R_{t}}-\frac{1}{R_{u}}$$

(5) $$\sigma =\frac{d}{R_{m}\cdot T\cdot W}$$

where *R_u_* is the resistance of ultra-pure water, *d* is the length of the membranes between inner sense electrodes, and *T* and *W* are the thickness and width of the AEMs, respectively. 

## 3. Results and Discussion

### 3.1. Chemical Structure

**Figure 2:**
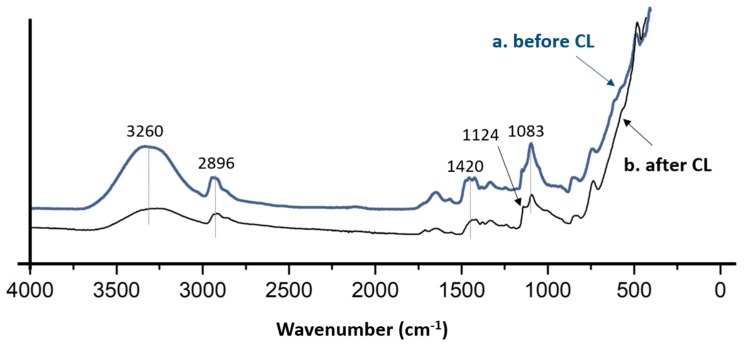
FTIR spectra of PVA/PADDA/NZ AEMs before and after cross-linking.

FTIR analysis was carried out to determine the main functional groups present in AEMs composite structures. Particularly the variations that occur before and after cross-linking were performed. Figure 2 shows the typical FTIR spectra of PVA/PDDA/NZ AEMs before and after introduced thermal-chemical cross-linking. The FTIR spectra demonstrate the wavenumber range from 4000 cm^−1^ to 400 cm^−1^, which comprises all potential frequencies of the infra-red vibrations for PVA, PDDA, and zirconia interactions. Both of the spectra display intensity at 3260 cm^−1^ and 1420 cm^−1^, as recognized as the stretching and bending vibration of –OH groups from PVA structure and the bound water throughout the measurement. The peak 2896 cm^−1^ emerges from the C–H stretch groups. The presence of a peak around 1083 cm^−1^ belongs to the stretching vibration of C–N groups from PDDA, which indicates the PDDA was successfully embedded into the PVA matrix. The decreasing of –OH groups intensity at 3240 cm^−1^ and the presence of a small peak at 1124 cm^−1^ corresponding to C–O–C groups specify that the reaction of chemical cross-linking between the –CHO functional groups of GA and the –H groups of PVA was successfully formed [32]. 

**Figure 3:**
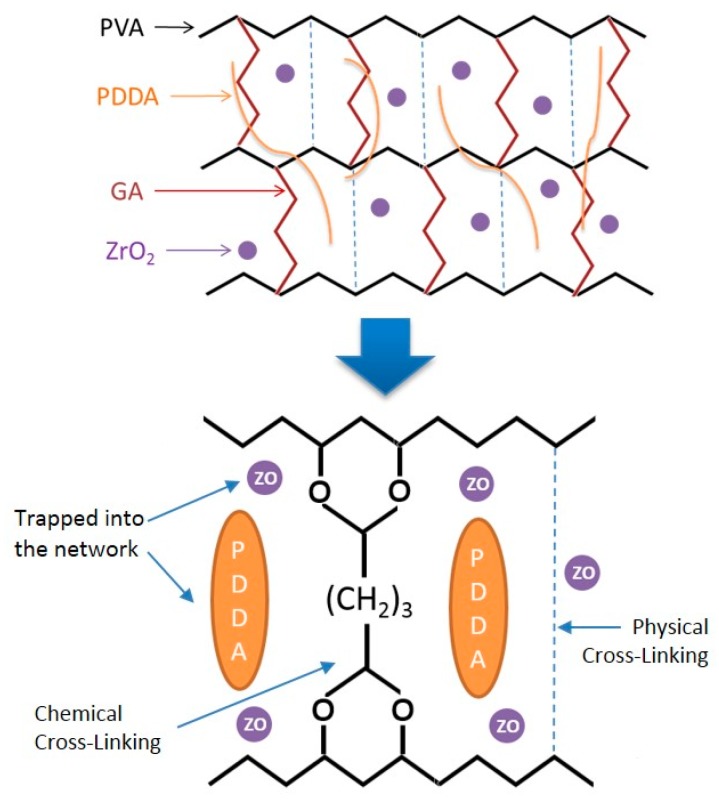
Model of the chemical structure of PVA/PDDA/NZ AEMs.

Figure 3 illustrates a proposed model of the chemical structure of AEMs based on the results of FTIR and literature [28,35]. The physical cross-linking between PVA chains is created by heat treatment (annealing). The heat treatment establishes hydrogen bonding between –OH groups of PVA chains, which promotes cross-linking to yield three-dimensional structures [36]. The chemical cross-linker induces covalent bonding between the –CHO functional groups of GA and the –H groups of PVA, which form a C–O–C bond. The PDDA and NZ inorganic nano-filler are trapped into the cross-linked PVA network as a typical interpenetrating polymer network.

### 3.2. Morphology

**Figure 4:**
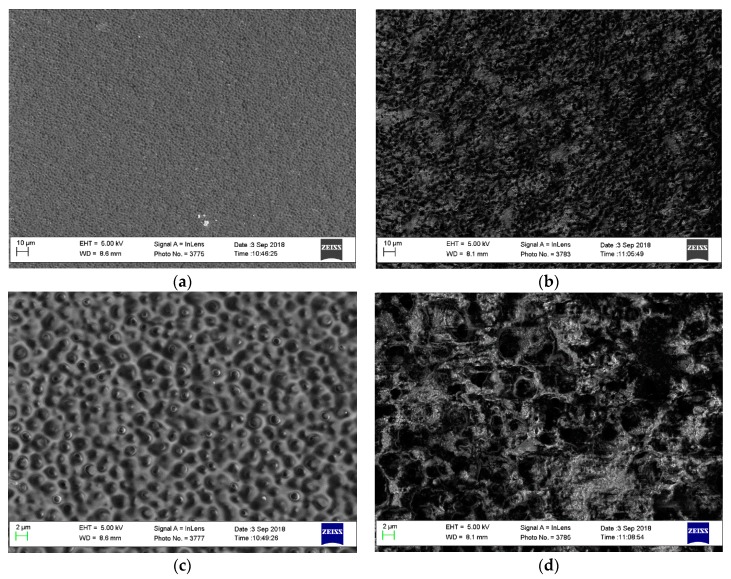
Pictures of the PVA/PDDA AEMs surface. (**a**) Without NZ, 1000×; (**b**) with NZ 2.5 wt.%, 1000×; (**c**) without NZ, 5000×; (**d**) with 2.5 wt.% NZ, 5000×.

Figure 4 depicts the surface morphologies of the PVA/PDDA and PVA/PDDA/2.5 wt.% NZ AEMs. From Figure 4a, it can be observed that the PVA/PDDA AEMs have a coarse surface structure, with small lump-like structures distributed homogeneously. Intermittently, small vesicles are seen at several lumps on Figure 4b. These presumably happened because of a typical interaction between PVA and PDDA causing the phase separation. This phenomenon leads to relatively loose structures and possibly establishes a microporous structure through swelling in water, which can enhance the membrane flexibility and water-holding capacity, expand the bulk of the membrane, affording more space for hydroxide transport in the membrane [28]. From Figure 4b,d, it can be seen that the coarse surface structure is improved after the addition of nano-zirconia in the PVA/PDDA membrane. The small vesicle at several lump surfaces disappeared, therefore the small cavity on the surface of the membrane is formed. This may have occurred due to the contact and interaction between zirconia filler and PVA/PDDA.

### 3.3. Thermal Analysis

**Figure 5:**
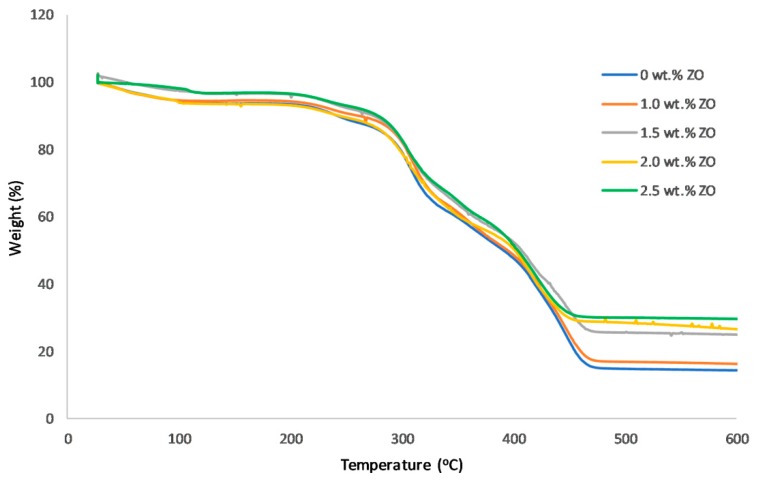
Thermogravigrams profiles of PVA/PDDA AEMs (PVA/PDDA = 1:0.5) with different NZ content.

The thermal stabilities of PVA/PDDA AEMs in addition to the different content of NZ are depicted in Figure 5. All the thermogravigrams illustrate three main weight loss steps at approximately 30–130 °C, 200–310 °C, and 320–470 °C. The first weight loss step indicates the release of water molecules in the AEMs and moisture absorbed from the atmosphere. The second weight loss step is potentially due to the decomposition of quaternary ammonium cationic groups, the cleavage of cross-linking bridges, and the breaking of some C–O and C–C bonds from PDDA and PVA, respectively [28]. The third weight loss step is associated with the decomposition of PVA and PDDA polymer backbones [37]. It can be observed that the addition of NZ reduces the weight loss of PVA/PDDA, illustrating that the composite AEMs acquire better thermal stability. Therefore, the thermogravimetric analysis results ensure the ample thermal stability of AEMs for the low-temperature fuel cells application.

### 3.4. Mechanical Properties

**Figure 6:**
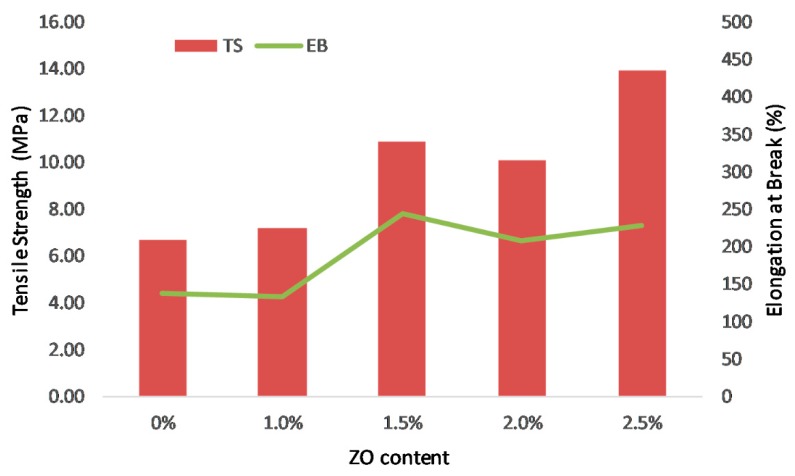
Tensile strength and elongation break of PVA/PADDA/NZ AEMs.

In order to withstand the pressing in the preparation of membrane electrode assembly, AEMs necessity achieves sufficient mechanical strength. Table 1 and Figure 6 show the mechanical properties, indicated by tensile strength and elongation at break, of the PVA/PDDA composite AEMs with different NZ content. Generally, the increase of the NZ content in the membranes is followed by the enhancement of the tensile strength and elongation at break. In conjunction with the increase of zirconia content, the membranes exhibited the maximum tensile strength of 13.96 MPa and elongation at break of 228.87% with zirconia content of 2.5% by mass. The improvement of mechanical properties is acquired from the strong interactions between the polymer matrix and the inorganic filler phase [30]. The addition of inorganic NZ nano-fillers into the polymer matrix can reduce the crystallinity of the polymer matrix, thus, it will enhance the amorphous phases of polymer matrix [29]. 

### 3.5. Water Uptake and Swelling Degree

The presence of water in the AEMs is essential for accomplishing high OH^−^ conductivity. Water clusters can provide transport channels for anions inside the anion-exchange membrane, in this way improving the ionic conductivity [32]. On the other hand, excessive water uptake can induce a severe swelling of the membrane, which leads to a decline of the dimensional stability and possibly restricts the preparation of membrane electrode assembly (MEA) by reducing contact between the active layer of the electrodes and the membrane [34]. 

**Figure 7:**
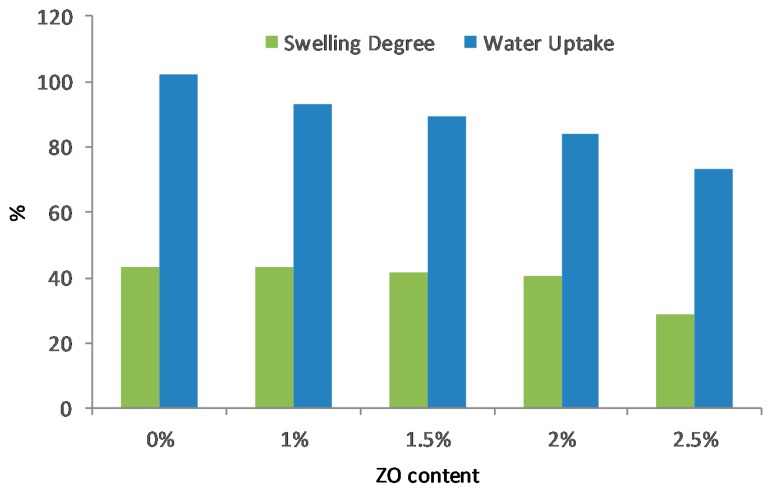
Water uptake and swelling degree of PVA/PADDA/NZ AEMs.

Table 1 and Figure 7 illustrate the water uptake (WU) and swelling degree (SD) of the PVA/PDDA membranes. The water uptake of PVA/PDDA membranes is 102%, which declines linearly with the addition of NZ 1%, 1.5%, 2%, 2.5%, resulting in a WU of 93%, 89%, 84%, and 73%, respectively. In the same way, the swelling degree result shows a similar occurrence. The swelling degree of pristine PVA/PDDA membranes is 43%. After the addition of 1% NZ, the swelling degree is still constant and slightly decreases to 42%, 41%, due to the addition of zirconia by 1.5% and 2% and sharply declines to 28% with the addition of 2.5 wt.% NZ. The reduction of WU and SD is probably due to the strengthening of interactions amongst the components in the AEMs, which leads to a more compact and denser micro-network structure. Therefore, the space occupied by water in the membrane decreases, which causes the decline of WU and SD [38]. This result is consistent with the SEM result (Figure 4), in which the small vesicles at several lumps due to phase separation as a result of PVA and PDDA interaction, disappear after the addition of NZ.

### 3.6. Ion-Exchange Capacity and OH^−^ Conductivity

**Figure 8:**
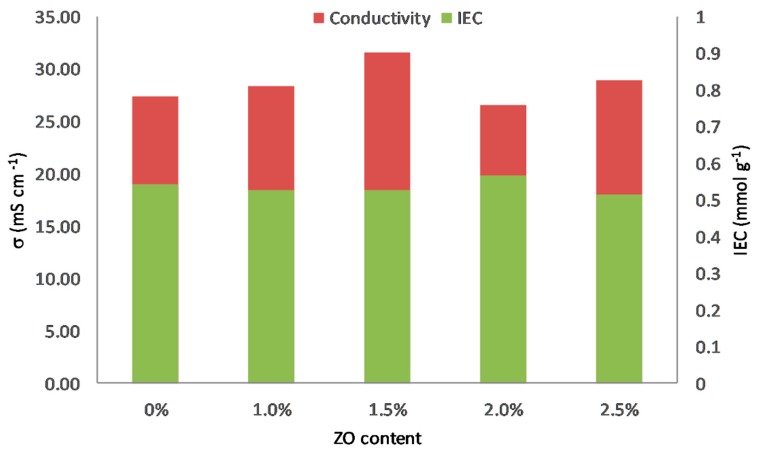
Ion exchange capacity and OH^−^ conductivity of PVA/PADDA/NZ AEMs.

Ion exchange capacity (IEC), an important parameter of the AEMs, exhibits the amount of ion-exchangeable groups in the AEMs, which is profoundly correspondent to the OH^−^ conductivity [33]. Table 1 and Figure 8 present the ion exchange capacity and OH^−^ conductivity of PVA/PADDA/NZ membranes at room temperature. The measured IEC values are within the narrow range of 0.52–0.56 mmol·g^−^^1^, which indicates that the addition of nano-zirconia filler did not significantly affect the IEC of membranes. The values of IEC might be considerably influenced by ion-conducting substance from PDDA, which has the equivalent ratio and concentration for each membrane.

The OH^−^ conductivity is one of the most essential properties of AEMs and is strongly attributed to the performance of APEFCs. It can be seen from Figure 5 that the OH^−^ conductivity increases from 27.4 mS·cm^−1^ of pristine PVA/PDDA membrane to 28.3 mS·cm^−1^ with the addition of 1.0 wt.% NZ. Therefore, the conductivity rises to 31.6 mS·cm^−1^ for 1.5 wt.% NZ contained PVA/PDDA membrane, which is the highest OH^−^ conductivity obtained from this work. The introduction of nano-ZrO_2_ fillers conceivably reduces the crystallinity and enhances the amorphous phases of the membrane matrix, leading to a rise of hydroxide ion conductivity of the AEMs [30]. However, following further inclusion of NZ, OH^−^ conductivity of PVA/PDDA/2 wt.% NZ and PVA/PDDA/2.5 wt.% NZ lessen to 26.5 mS·cm^−1^ and 28.9 mS·cm^−1^, respectively. The decrease of OH^−^ conductivity could be due to the excessive number of NZ in the polymer matrix leading to a reduction of the free volume of the membrane, and losses of the space occupied by water. According to the Grotthuss mechanism, water clusters can provide transport channels for anions inside the AEMs, leading to an improvement of the OH^−^ conductivity [39]. 

Due to the contact of AEMs with the ambient air during the OH^−^ conductivity measurement, a carbonation process may partially occur. The result emerges in which OH^−^ as a counter-anion of quaternary ammonium functional groups in PDDA with CO_2_ present in the air to form CO_3_^2^^−^ and HCO^3^^−^ anions according to reaction 6 and 7, respectively [40]. The main effect of this carbonation process is a significant decrease in the effective anion conductivity in the AEM [41].

OH^−^ + CO_2_ ⇌ HCO^3^^−^ (6)

HCO^3^^−^ + OH^−^ ⇌ CO_3_^2^^−^ + H_2_O (7)

The occurrence of CO_3_^2^^−^ and HCO^3^^−^ anions is unfavorable because of reducing the effective conductivity of the AEMs. This phenomenon occurs because these anions have a larger ionic radius than hydroxide, leading to lower diffusion coefficients and lower mobility [40]. According to this potential carbonation process, the OH^−^ conductivity presented in Table 1 and Figure 8 conceivably become lower than the real value of pure OH^−^ form. However, comparing the resulting IEC and OH^−^ conductivity with the published results of PVA-based AEMs (Table 2), PVA/PDDA/NZ AEMs provide higher OH^−^ conductivity, although they have a lower IEC.

## 4. Conclusions

The novel PVA/PDDA/NZ composite AEM was successfully prepared by a blending, cross-linking, and solution casting method. FTIR spectroscopy reveals the cross-linking bridge between the –OH groups of PVA and the –CHO groups of glutaraldehyde. The three major weight loss stages from the thermogravigrams confirm the sufficient thermal stability for the low-temperature fuel cell applications. The addition of NZ significantly improves the mechanical properties of AEMs and becomes one the main advantages of this work. The membrane with the 2.5 wt.% NZ content achieved the highest mechanical properties with the maximum tensile strength and elongation of 13.96 MPa and 228.87%, respectively. The introduction of NZ decreases the water uptake and swelling degree of AEMs. Highest OH^−^ conductivity of 31.6 mS·cm^−1^ at ambient temperature was obtained for a polymer composition PVA/PDDA/1.5 wt.% NZ with IEC of 0.52 mmol·g^−1^. Incorporation of nano-zirconia is confirmed to be an excellent scheme to enhance the electrochemical and physicochemical properties of PVA/PDDA membranes. In conclusion, this work demonstrates that the developed PVA/PDDA/NZ could offer new prospects for use in alkaline polymer electrolyte fuel cells.

## Figures and Tables

**Table 1:** Physicochemical parameters of PVA/PDDA/NZ membranes.

NZ Content (%)	IEC (mmol·g^−1^)	WU (%)	SD (%)	TS (MPa)	*E_b_* (%)	σ (mS·cm^−1^)
0	0.54	102	43	6.73	138.32	27.3
1.0	0.52	93	43	7.21	134.34	28.3
1.5	0.52	89	42	10.87	245.27	31.6
2.0	0.57	84	40	10.13	207.64	26.5
2.5	0.52	73	29	13.96	228.87	28.9

**Table 2:** Ion exchange capacity and OH^−^ conductivity reported in the literature for PVA based AEMs at 25–30 °C.

Materials	IEC (mmol·g^−1^)	Conductivity (mS·cm^−1^)	References
QPVA/HDT	0.73	4.84 (30 °C)	[42]
QPVA/CS/MoS_2_	0.89	32 (25 °C)	[43]
QPVA/QCS	1.75	16 (25 °C)	[34]
CS/PVA/GO	0.38	0.19 (25 °C)	[44]
PVA/PDDA	0.85	25 (25 °C)	[35]
PVA/PAADDA	1.63	3 (25 °C)	[45]
QPVA/Q-SiO_2_	0.65	2.37 (25 °C)	[38]
PVA/PDDA/MWNT	0.89	30.3 (30 °C)	[28]
PVA/PDDA/ZrO_2_	0.54	31.6 (RT)	This work

## References

[1] Dai W., Wang H., Yuan X., Martin J.J., Yang D. (2009). A review on water balance in the membrane electrode assembly of proton exchange membrane fuel cells. Int. J. Hydrogen Energy.

[2] Fang J., Wu Y., Zhang Y., Lyu M., Zhao J. (2015). Novel anion exchange membranes based on pyridinium groups and fluoroacrylate for alkaline anion exchange membrane fuel cells. Int. J. Hydrogen Energy.

[3] Couture G., Alaaeddine A., Boschet F., Ameduri B. (2011). Polymeric materials as anion-exchange membranes for alkaline fuel cells. Prog. Polym. Sci..

[4] Wang Y.-J., Qiao J., Baker R., Zhang J. (2013). Alkaline polymer electrolyte membranes for fuel cell applications. Chem. Soc. Rev..

[5] Iravaninia M., Rowshanzamir S. (2015). Polysulfone-based Anion Exchange Membranes for Potential Application in Solid Alkaline Fuel Cells. J. Renew. Energy Environ..

[6] Hu B., Miao L., Zhao Y., Lü C. (2017). Azide-assisted crosslinked quaternized polysulfone with reduced graphene oxide for highly stable anion exchange membranes. J. Memb. Sci..

[7] Xue J., Liu L., Liao J., Shen Y., Li N. (2017). UV-crosslinking of polystyrene anion exchange membranes by azidated macromolecular crosslinker for alkaline fuel cells. J. Memb. Sci..

[8] Liu W., Liu L., Liao J., Wang L., Li N. (2017). Self-crosslinking of comb-shaped polystyrene anion exchange membranes for alkaline fuel cell application. J. Memb. Sci..

[9] Xiong Y., Liu Q.L., Zhang Q.G., Zhu A.M. (2008). Synthesis and characterization of cross-linked quaternized poly(vinyl alcohol)/chitosan composite anion exchange membranes for fuel cells. J. Power Sources.

[10] Gong Y., Liao X., Xu J., Chen D., Zhang H. (2016). Novel anion-conducting interpenetrating polymer network of quaternized polysulfone and poly(vinyl alcohol) for alkaline fuel cells. Int. J. Hydrogen Energy.

[11] Ouadah A., Xu H., Luo T., Gao S., Wang X., Fang Z., Jing C., Zhu C. (2017). A series of poly(butylimidazolium) ionic liquid functionalized copolymers for anion exchange membranes. J. Power Sources.

[12] Hu Q., Shang Y., Wang Y., Xu M., Wang S., Xie X., Liu Y., Zhang H., Wang J., Mao Z. (2012). Preparation and characterization of fluorinated poly(aryl ether oxadiazole)s anion exchange membranes based on imidazolium salts. Int. J. Hydrogen Energy.

[13] Shi Q., Chen P., Zhang X., Weng Q., Chen X., An Z. (2017). Synthesis and properties of poly(arylene ether sulfone) anion exchange membranes with pendant benzyl-quaternary ammonium groups. Polym. (United Kingdom).

[14] Hu E.N., Lin C.X., Liu F.H., Wang X.Q., Zhang Q.G., Zhu A.M., Liu Q.L. (2018). Poly(arylene ether nitrile) anion exchange membranes with dense flexible ionic side chain for fuel cells. J. Memb. Sci..

[15] Xu J., Liu B., Luo X., Li M., Zang H., Zhang H., Wang Z. (2017). Construction of ion transport channels by grafting flexible alkyl imidazolium chain into functional poly(arylene ether ketone sulfone) as anion exchange membranes. Int. J. Hydrogen Energy.

[16] Omasta T.J., Wang L., Peng X., Lewis C.A., Varcoe J.R., Mustain W.E. (2017). Importance of balancing membrane and electrode water in anion exchange membrane fuel cells. J. Power Sources.

[17] Cheng J., He G., Zhang F. (2015). A mini-review on anion exchange membranes for fuel cell applications: Stability issue and addressing strategies. Int. J. Hydrogen Energy.

[18] Li N., Yan T., Li Z., Thurn-Albrecht T., Binder W.H. (2012). Comb-shaped polymers to enhance hydroxide transport in anion exchange membranes. Energy Environ. Sci..

[19] Dang H.S., Weiber E.A., Jannasch P. (2015). Poly(phenylene oxide) functionalized with quaternary ammonium groups via flexible alkyl spacers for high-performance anion exchange membranes. J. Mater. Chem. A.

[20] Nuñez S.A., Capparelli C., Hickner M.A. (2016). N-Alkyl Interstitial Spacers and Terminal Pendants Influence the Alkaline Stability of Tetraalkylammonium Cations for Anion Exchange Membrane Fuel Cells. Chem. Mater..

[21] Wang C., Shen B., Xu C., Zhao X., Li J. (2015). Side-chain-type poly(arylene ether sulfone)s containing multiple quaternary ammonium groups as anion exchange membranes. J. Memb. Sci..

[22] Zhang J., Liu L., Ma C., Liu Y., Qiao J. (2013). Poly (vinyl alcohol)/sulfosuccinic acid (PVA/SSA) as proton-conducting membranes for fuel cells: Effect of cross-linking and plasticizer addition. ECS Trans..

[23] Zakeri M., Abouzari-Lotf E., Nasef M.M., Ahmad A., Ripin A., Ting T.M., Sithambaranathan P. (2018). Preparation and characterization of highly stable protic-ionic-liquid membranes. Int. J. Hydrogen Energy.

[24] Vinodh R., Sangeetha D. (2013). Efficient utilization of anion exchange composites using silica filler for low temperature alkaline membrane fuel cells. Int. J. Plast. Technol..

[25] Yang C.C., Chiu S.J., Chien W.C., Chiu S.S. (2010). Quaternized poly(vinyl alcohol)/alumina composite polymer membranes for alkaline direct methanol fuel cells. J. Power Sources.

[26] Derbali Z., Fahs A., Chailan J.F., Ferrari I.V., Di Vona M.L., Knauth P. (2017). Composite anion exchange membranes with functionalized hydrophilic or hydrophobic titanium dioxide. Int. J. Hydrogen Energy.

[27] Li X., Tao J., Nie G., Wang L., Li L., Liao S. (2014). Cross-linked multiblock copoly(arylene ether sulfone) ionomer/nano-ZrO_2_ composite anion exchange membranes for alkaline fuel cells. RSC Adv..

[28] Zhou T., Wang M., He X., Qiao J. (2019). Poly(vinyl alcohol)/Poly(diallyldimethylammonium chloride) anion-exchange membrane modified with multiwalled carbon nanotubes for alkaline fuel cells. J. Mater..

[29] Vinodh R., Purushothaman M., Sangeetha D. (2011). Novel quaternized polysulfone/ZrO2 composite membranes for solid alkaline fuel cell applications. Int. J. Hydrogen Energy.

[30] Li X., Yu Y., Meng Y. (2013). Novel quaternized poly(arylene ether sulfone)/nano-ZrO2 composite anion exchange membranes for alkaline fuel cells. ACS Appl. Mater. Interfaces.

[31] Zhou T., Zhang J., Jingfu J., Jiang G., Zhang J., Qiao J. (2013). Poly(ethylene glycol) plasticized poly(vinyl alcohol)/poly(acrylamide-co- diallyldimethylammonium chloride) as alkaline anion-exchange membrane for potential fuel cell applications. Synth. Met..

[32] Zhang J., Qiao J., Jiang G., Liu L., Liu Y. (2013). Cross-linked poly(vinyl alcohol)/poly (diallyldimethylammonium chloride) as anion-exchange membrane for fuel cell applications. J. Power Sources.

[33] Yuan Y., Shen C., Chen J., Ren X. (2018). Synthesis and characterization of cross-linked quaternized chitosan/poly(diallyldimethylammonium chloride) blend anion-exchange membranes. Ionics (Kiel).

[34] Feketeföldi B., Cermenek B., Spirk C., Schenk A., Grimmer C., Bodner M., Koller M., Ribitsch V., Hacker V. (2016). Chitosan-Based Anion Exchange Membranes for Direct Ethanol Fuel Cells. J. Membr. Sci. Technol..

[35] Qiao J., Zhang J., Zhang J. (2013). Anion conducting poly(vinyl alcohol)/poly(diallyldimethylammonium chloride) membranes with high durable alkaline stability for polymer electrolyte membrane fuel cells. J. Power Sources.

[36] Miraftab M., Saifullah A.N., Çay A. (2015). Physical stabilisation of electrospun poly(vinyl alcohol) nanofibres: Comparative study on methanol and heat-based crosslinking. J. Mater. Sci..

[37] Qiao J., Fu J., Liu L., Liu Y., Sheng J. (2012). Highly stable hydroxyl anion conducting membranes poly(vinyl alcohol)/poly(acrylamide-co-diallyldimethylammonium chloride) (PVA/PAADDA) for alkaline fuel cells: Effect of cross-linking. Int. J. Hydrogen Energy.

[38] Yang C.C., Chiu S.S., Kuo S.C., Liou T.H. (2012). Fabrication of anion-exchange composite membranes for alkaline direct methanol fuel cells. J. Power Sources.

[39] Peighambardoust S.J., Rowshanzamir S., Amjadi M. (2010). Review of the proton exchange membranes for fuel cell applications. Int. J. Hydrogen Energy.

[40] Ziv N., Dekel D.R. (2018). A practical method for measuring the true hydroxide conductivity of anion exchange membranes. Electrochem. Commun..

[41] Suzuki S., Muroyama H., Matsui T., Eguchi K. (2013). Influence of CO2 dissolution into anion exchange membrane on fuel cell performance. Electrochim. Acta.

[42] Hari Gopi K., Bhat S.D. (2018). Anion exchange membrane from polyvinyl alcohol functionalized with quaternary ammonium groups via alkyl spacers. Ionics (Kiel).

[43] Jiang X., Sun Y., Zhang H., Hou L. (2018). Preparation and characterization of quaternized poly(vinyl alcohol)/chitosan/MoS2composite anion exchange membranes with high selectivity. Carbohydr. Polym..

[44] García-Cruz L., Casado-Coterillo C., Irabien Á., Montiel V., Iniesta J. (2016). High Performance of Alkaline Anion-Exchange Membranes Based on Chitosan/Poly (vinyl) Alcohol Doped with Graphene Oxide for the Electrooxidation of Primary Alcohols. C.

[45] Qiao J., Fu J., Liu L., Zhang J., Xie J., Li G. (2012). Synthesis and properties of chemically cross-linked poly(vinyl alcohol)-poly(acrylamide-co-diallyldimethylammonium chloride) (PVA-PAADDA) for anion-exchange membranes. Solid State Ionics.

